# Supplementary material for: Identification of photoperiod-induced specific miRNAs in the adrenal glands of Sunite sheep (Ovis aries)
Source: Front Vet Sci. 2022 Jul 22;9:888207. doi: 10.3389/fvets.2022.888207 (PMC9354845; doi:10.3389/fvets.2022.888207)
Supplement: Supplementary file 1 [file Data_Sheet_1.ZIP › Supplementary file/miRDeep2 file.zip!/miRDeep2 file/Know_Structure/SP42A2/oar-mir-541.pdf]

miRBase precursor : oar-mir-541
Total read count : 262
oar-miR-541-5p read count : 246
oar-miR-541-3p read count : 16
remaining reads : 0

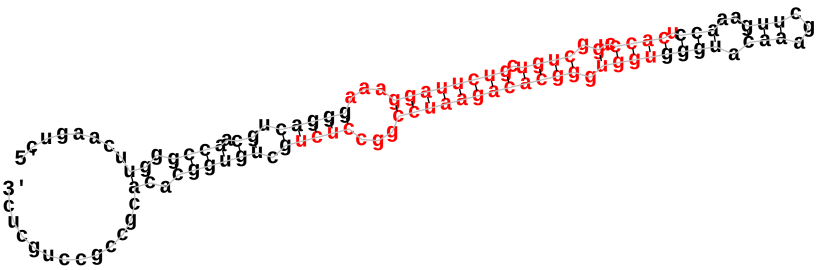

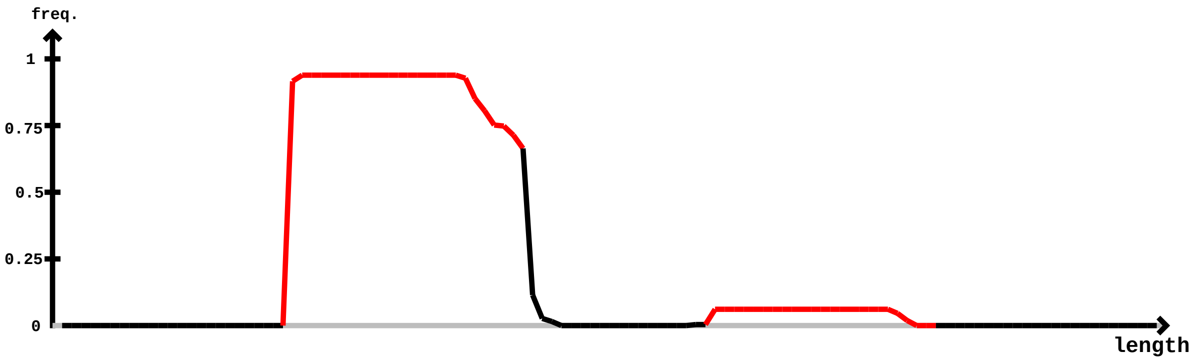


```
                                                                 oar-miR-541-3p
                           oar-miR-541-5p
5'- cugaacuugggccaacgucagggaaaggauucugcugucggucccacuccaaaguucgaaacauggguggugggcacagaaauccggccucugcuguggcacacgccgccugcuc  -3'  exp
    ........((((..(((.(((((...((((((((.((((.(..((((.(((..(((...))).)))))))).))))))))))))...))))).)))(((....)))))))....  reads  mm  sample
    .......................aaaggauucugcugucgg..........................................................................  3      0   seq
    .......................aaaggauucugcugucggu.........................................................................  20     0   seq
    .......................aaaggauucugcugucgguc........................................................................  12     0   seq
    .......................aaaggauucugcugucggucc.......................................................................  14     0   seq
    .......................aaaggauucugcugucgguccca.....................................................................  9      0   seq
    .......................aaaggauucugcugucggucccac....................................................................  12     0   seq
    .......................aaaggauucugcugucggucccacu...................................................................  141    0   seq
    .......................aaaggauucugcugucggucccacuc..................................................................  23     0   seq
    .......................aaaggauucugcugucggucccacucc.................................................................  2      0   seq
    .......................aaaggauucugcugucggucccacucca................................................................  4      0   seq
    ........................aaggauucugcugucgguccc......................................................................  1      0   seq
    ........................aaggauucugcugucggucccac....................................................................  1      0   seq
    ........................aaggauucugcugucggucccacu...................................................................  3      0   seq
    ........................aaggauucugcugucggucccacucc.................................................................  1      0   seq
    .................................................................gguggugggcacagaaauccggc...........................  1      0   seq
    ...................................................................uggugggcacagaaauccgg............................  4      0   seq
    ...................................................................uggugggcacagaaauccggc...........................  6      0   seq
    ...................................................................uggugggcacagaaauccggcc..........................  5      0   seq
```
